# Protocol for ultrasound-guided injection into the murine portal vein to initiate liver metastasis

**DOI:** 10.1016/j.xpro.2026.104670

**Published:** 2026-07-04

**Authors:** Nicole Henning, Fred Roberts, Virginia Spanoudaki

**Affiliations:** 1 Preclinical Imaging and Testing Facility, Koch Institute for Integrative Cancer Research, Massachusetts Institute of Technology, Cambridge, MA 02142, USA; 2 EMIT Imaging, Baltimore, MD, 21201, USA Formerly of Fujifilm VisualSonics Inc., Toronto, ON M4N 3N1, Canada

**Keywords:** Cancer, Model Organisms, Organoids, Biotechnology and bioengineering

## Abstract

Here, we present a non-surgical protocol for modeling murine liver metastasis by disseminating injected cells or compounds into the murine portal vein. We describe steps for identifying the portal vein and other vital structures adjacent to and within the liver by ultrasound, determining an optimal route to avoid vital structures, and injecting into the portal vein. We also report on the findings for reproducibility, adverse effects, and histopathological findings.

## Highlights

• Instructions for modeling murine liver metastasis using ultrasound-guided injection
• Steps to configure ultrasound and injection systems and prepare and position mouse
• Guidance to visualize and identify portal vein and define a transhepatic needle route
• Procedures to confirm transverse vessel placement and optimize view prior to injection

## Abstract (editor highlights)

Publisher’s note: Undertaking any experimental protocol requires adherence to local institutional guidelines for laboratory safety and ethics.

## Graphical abstract

**Figure:**
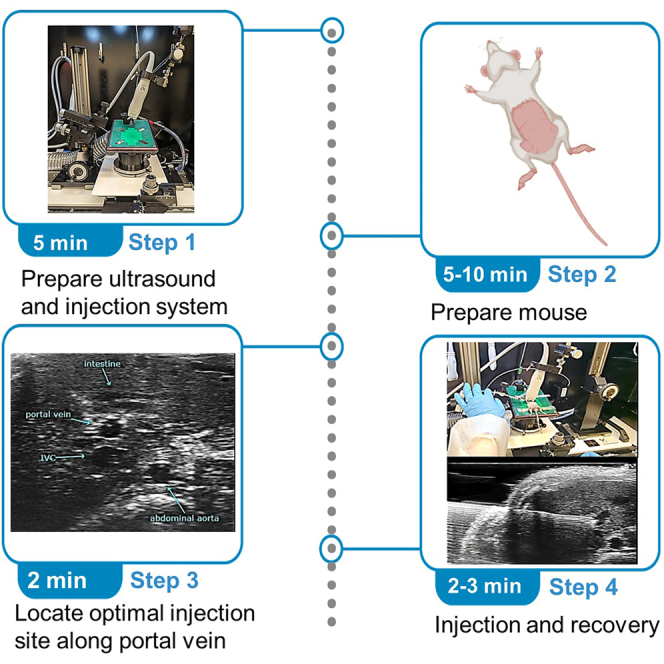

## Before you begin

The protocol below describes the specific steps for injecting into the portal vein by ultrasound guidance (USGI) within the liver of 6–15 weeks old male or female (17–21 g) mice of various immuno-competent and immuno-compromised strains. This protocol will describe the technique using an injection of sterile saline as a test injectate. In expected outcomes we report the feedback from researchers that have utilized the described injection protocol using cells with the goal of modeling liver metastasis.

### Innovation

This protocol offers several advancements over previously reported methods.^1^ First, it uses anatomical landmarks that are simpler to identify and distinguish, namely the portal vein in the liver distinguished from the inferior vena cava and the abdominal aorta. Previous methods require advanced technical expertise to identify multiple organs and vessels, then tracing the vessels to distinguish them. Second, the current method opts for a needle path directly into the liver tissue avoiding other vital organs which are not intended for injection. Thus, avoiding cell seeding outside of the liver. Moreover, the liver tissue is easier to penetrate than the pancreas. Third, this protocol uses a commercially available attachment to allow for flexibility in visualizing the needle instead of 3D printing a needle fixation device. Additionally, the attachment aids in needle placement verification, as it enables visualization of a proximal portion of the vessel relative to the needle within it.

### Institutional permissions

This procedure was performed in the AAALACi accredited imaging suite of Massachusetts Institute of Technology’s Preclinical Imaging and Testing Core facility (RRID:SCR_026699) or in one of the adjacent housing areas when working with animals and inoculants which require a biosafety cabinet protection. All described steps below were performed under protocols approved by the Committee on Animal Care of the Massachusetts Institute of Technology.

**Figure 1:**
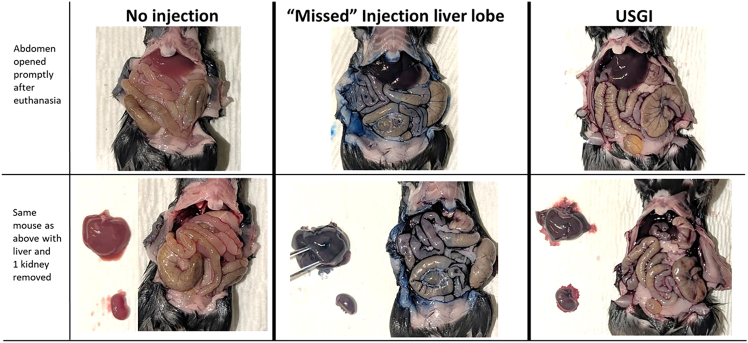
Methylene Blue Dye Injections Injection of a 1% methylene blue dye immediately followed by euthanasia and necropsy. Top row images are taken just after opening the abdomen and bottom images are same mouse with liver and one kidney removed. Columns from left to right: Left is a non-injected naive mouse, center is an example of a missed injection into the liver lobe, and right is an example of a successful USGI portal vein injection. Of note is that the blue dye has darkened the liver in the successful example versus the naïve mouse, whereas in the missed injection a majority of the peritoneal viscera has a blue tint.

## Key resources table

**Table:** 

REAGENT or RESOURCE	SOURCE	IDENTIFIER
**Chemicals, peptides, and recombinant proteins**		

Sterile saline: 0.9% Sodium Chloride Injection, USP	Hospira	00409488810
Isoflurane	Medline	DVM001325EA
Depilatory hair cream: Nair® or Veet®	NA	NA

**Experimental models: Organisms/strains**		

Mouse: various strains, aged 6–15 weeks, male and female	Commercial vendors for specific pathogen free mice	

**Other**		

Isoflurane vaporizer	Vet Equip Inc.	911103; http://www.vetequip.com/default.aspx
Animal fur shaver: Wahl Peanut or similar	NA	NA
Integrated rail system with image guided injection system	VisualSonics	https://www.visualsonics.com/product/accessories/imaging-stations
Micro-manipulator ultrasound transducer clamp (Y-axis transducer adjuster) (henceforth referred to as transducer micro-manipulator)	VisualSonics	https://www.visualsonics.com/contact-us/contact-sales
Mouse imaging platform	VisualSonics	https://www.visualsonics.com/contact-us/contact-sales
Advanced Physiological Monitoring Unit “THM-150” or similar	VisualSonics	https://www.visualsonics.com/contact-us/contact-sales
MX550S (40MHz) ultrasound transducer or similar	VisualSonics	https://www.visualsonics.com/content/browse-all-transducers
Vevo 3100 Imaging System (no longer offered) or similar	VisualSonics	RRID:SCR_022152; https://www.visualsonics.com/product/imaging-systems/vevo-3100
Transpore™ Surgical Tape, 1527-0, 1/2 in X 10 yd	3 M™	30707387794560
Aquasonic® 100 Trans Gel or similar	Parker Laboratories	PLI 01-50
Alcohol swab	BD	326895
Disposable Tuberculin Syringe with Slip Tip	BD	309659
Accu-Needle 33G × ½”	Acuderm	NP335; https://www.acuderm.com/

## Materials and equipment

***Alternatives:*** A portable monitor (ViewSonic VA1655–15.6” Portable 1080p IPS Monitor with USB C and mini-HDMI or similar) can be set up behind the integrated rail system to aid the injectionist ergonomically and to aid in easily viewing the ultrasound image while their hands are manipulating items for injection. The Vevo 3100 has a display port connection (HDMI) that the portable monitor can be connected to.

***Alternatives:*** An injection of a 1% methylene blue (Sigma-Aldrich or similar) dye immediately followed by euthanasia and necropsy can be used to validate the technique. The whole liver should appear colored and no dye should be found in the peritoneal space. See Figure 1 “Methylene blue dye injections” for examples of a successful USGI portal vein injection, a missed injection into the liver lobe, and a naive mouse.

***Alternatives:*** When piloting tumor cell injections, we suggest experimenting with several different cell concentrations for each cell line to reach the optimal concentration for the desired model.

***Alternatives:*** Ultrasound gel can be aliquoted (∼40mL) into 50mL conical tubes then centrifuged at 750–850 g for 2 min to remove bubbles. Gardening hand pruning shears can be used to remove part of the tip of the conical tube. After removing the tube cap, a wadded paper towel inserted acts as a plunger to push the ultrasound gel out the opened tip-end for dispensing.

## Step-by-step method details

### Preparing the ultrasound setup and the mouse

**Timing: 5 min**

During this step prepare the ultrasound imaging set up and the mouse. See Figure 2 “Procedure Set Up” for a labeled overview of system components: the image guided injection system, the mouse imaging platform, the mounted transducer and their set-up relative to each other. See Figure 3 “Injection System Labeled” (Image repurposed with permission from previous publication^2^) for a labeled close up of the image guided injection system. See Methods Video S1 “Mouse Placement” for placement of the mouse in steps 8 and 9.

1. Select the mouse abdominal application on the ultrasound system and open the image width fully and the image depth between 10 and 12mm. Place the focal point of the ultrasound at 7mm.
2. Ensure the micro-manipulator transducer clamp is set to be adjustable in the Y-axis, then mount the MX550S transducer on the clamp.

***Note:*** The Micro-manipulator ultrasound transducer clamp (Y-axis transducer adjuster) will henceforth be referred to as “transducer micro-manipulator”

***Note:*** The orientation notch on the transducer should match the image on the monitor (usually to the left side). See Figure 2.

3. Position the transducer transverse to the imaging platform then adjust the transducer arm joint to angle the transducer about 30° away from the vertical towards the injection mount.

***Note:*** The adjusted transducer angle allows for a wider opening for the needle to pass under the transducer. Ensure the probe remains transverse to the imaging platform when making the adjustment.

4. Align injection system components.
  a. Ensure the injection system is perpendicular to the imaging platform.
  b. Adjust the syringe mount angle to approximately 30° below the horizontal plane. (The horizontal plane is the work surface that the integrated rail and image guided injection systems are sitting on).
  c. Ensure the needle driver is fully retracted and the ruler is at zero.
  d. Ensure the injection system Z- and Y-axis fine adjustment knobs to be in the middle of their movement range (12.5mm for Z-axis, 10mm for Y-axis).
5. Adjust the imaging platform X- and Y-axis fine adjustment knobs to be in the middle of their movement range (12.5mm for X-axis, 10mm for Y-axis).
6. Turn on the supplemental heat to the imaging platform on the THM-150 and set the platform temperature at 37°C.
7. Under isoflurane anesthesia shave and depilate the mouse on the ventral surface across its upper right abdominal quadrant.

**Pause point:** Fur removal can occur up to 24 h ahead of the injection procedure.

8. Position the mouse and maintain the isoflurane level at 2% at the imaging platform nose cone.
  a. Place mouse supine on the imaging platform with all four limbs slightly outstretched.
  b. Apply tape across the front limbs including across the lower jaw to the nose cone.
  c. Apply tape across the pelvis.
9. Tilt the imaging platform slightly (approximately 15°) down on the caudal side.

***Note:*** This tilt allows the abdominal organs to be pulled downward and away from the liver somewhat.

**Figure 2:**
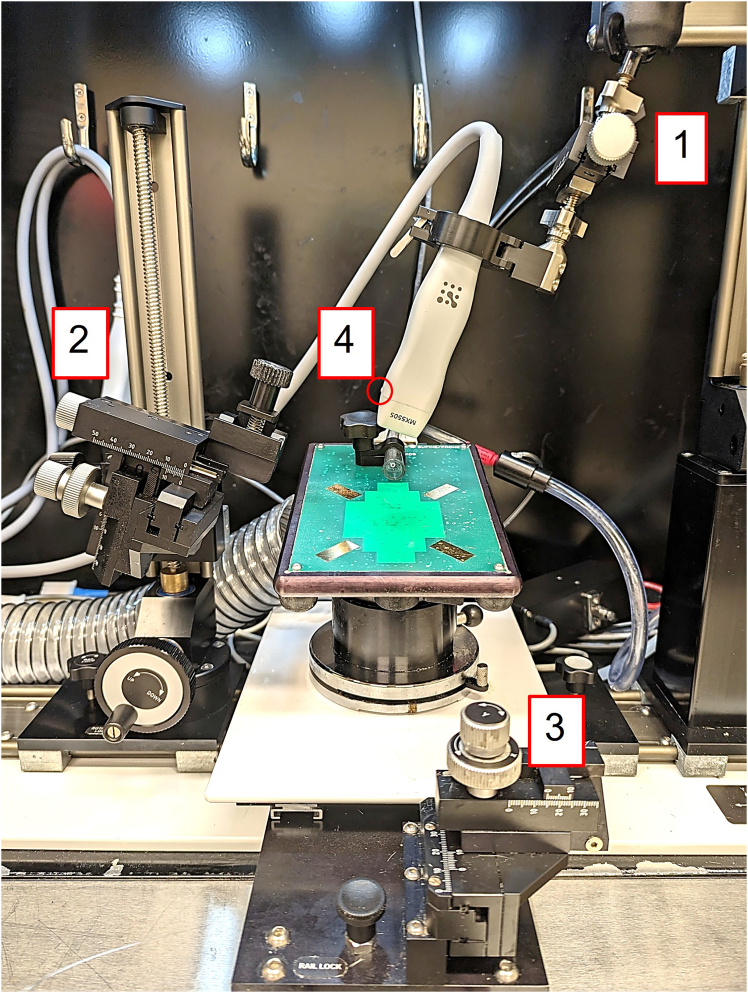
Procedure Set Up 1. Micro-manipulator ultrasound transducer clamp (Y-axis transducer adjustor). 2. Image guided injection system. 3. Imaging platform X- and Y-axis fine adjustment knobs. 4. Transducer orientation notch.

**Figure 3:**
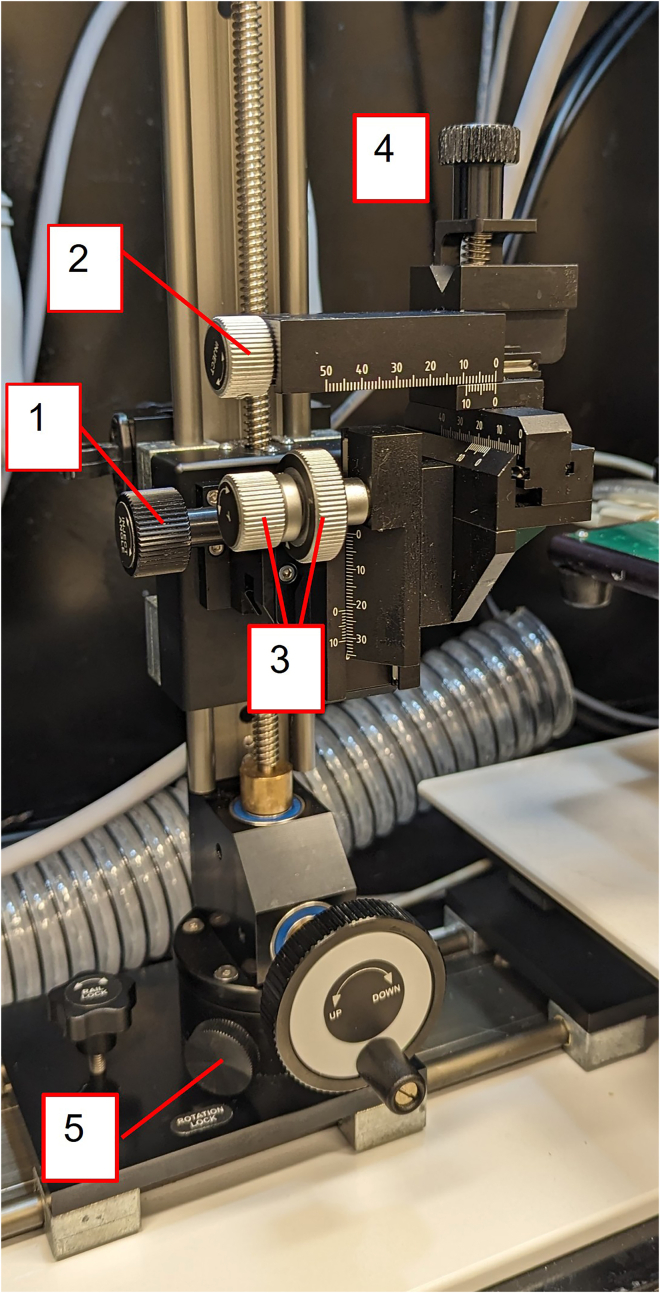
Injection System Labeled (Image repurposed with permission from previous publication^2^) 1. Syringe mount angle adjustor. 2. Needle driver knob. 3. Injection system Y- and Z-axis fine adjustment knobs. 4. Syringe mount. 5. Injection system rotation lock.

Methods Video S1. Mouse Placement, related to step #8 and 9

### Locate and inject into the portal vein within the liver

**Timing: 5–10 min**

During these steps locate the portal vein within the liver and differentiate it from other nearby vessels. The optimal site along the portal vein is where it is as superficial to the ventral surface as possible and there will be no bowel present along the needle path to the vessel. See Methods Video S2 “Vessels in Liver Identified” which identifies these vessels and bowel. Next, proceed with the injection and recover the mouse. See Methods Video S3 “Dual Synchronized Video Procedure” for a side-by-side video from the sonographer’s point-of-view synchronized with the ultrasound image produced following the stepwise directions #12–22. Note that, for brevity, the 30 s wait with the needle remaining in place post-injection in step 22 b has not been included in this video.

10. Mount the syringe in the syringe mount so the syringe scale is visible and the needle bevel is facing up. Then wipe the needle with an alcohol swab.
11. Apply a fair amount of ultrasound gel across the prepared area as well as to flow over the mouse’s right flank.
12. Lower the transducer onto the prepared area with roughly 1 to 2mm of gel between the transducer face and the skin line.
13. Visualize the portal vein deep in the liver.

***Note:*** The portal vein is distinguished on ultrasound by the hyperechoic (bright white) border surrounding it (assuming no other pathology is present in the liver, such as fibrosis) and the portal vein does not pulse. The inferior vena cava (IVC) and the abdominal aorta are located dorsally to the portal vein and the abdominal aorta has a visible pulse. The portal vein is the most ventral of the three vessels.

***Optional:*** Adjust the gain accordingly to have adequate image quality to identify the portal vein and other vessels.

14. Use the Y-axis fine adjustment knob of the imaging platform to scan several millimeters cranio-caudal to identify an optimal needle path.

**CRITICAL:** Identify an area of the liver with a relatively short distance from the skin line to the portal vein and no bowel is present along the needle path as identified by peristaltic movements.

15. Slide the injection system towards the mouse to bring the needle within the ultrasound gel on the flank but not quite touching the mouse.
16. Release the knob on the injection system that locks the rotation of the system assembly and then retighten to set to the default perpendicular position.

***Note:*** This will allow for the injection system to be reset to the default positioning since changing of the syringe and other adjustments can cause misalignment of the injection system.

17. Using the injection system Y-axis fine adjustment knob bring the length of the needle to be in plane and visualized by the ultrasound transducer.
18. Use the needle to create skin tension for a smooth puncture and align the needle for insertion:
  a. Using the injection system Z-axis fine adjustment knob, adjust the needle height to visualize the needle tip high up on the flank (approximately 2mm below the horizontal ventral surface).
  b. Slide the injection system in so the needle applies only enough pressure to cause a small dimple in the skin surface without puncturing it.
  c. Adjust the needle down 2–4mm using the Z-axis fine adjustment knob to be approximately lined up for insertion into the portal vein.

***Optional:*** The Needle Guide tool can help the injectionist visualize an estimate of the needle path atop the ultrasound image. To use this, activate the tool on the ultrasound to display.

19. Place one or several right-hand fingers on the mouse’s left side (contralateral side) in a supportive position to counteract any force during needle insertion. Do not push on the mouse.
20. Insert the needle into the liver with slow and steady clockwise turns of the needle driver knob.

**CRITICAL:** If the needle tip and bevel go out of the imaging plane, and are no longer visible, adjust the transducer position using the knob on the transducer micro-manipulator.

***Note:*** Adjustments to the needle angle (with the syringe mount angle adjustment knob) or to the ventral/dorsal height position (with the Z-axis fine adjustment knob) may be needed to realign the needle for entry into the vein.

21. Continue to drive the needle forward until it is within the portal vein.

***Note:*** The walls of the portal vein are flexible and require tension for the needle to puncture. This will appear as the wall of the vein bending concave to the pressure of the needle until it is touching the opposite wall of the vein and then it releases over the needle as it is punctured. This may also result in puncturing through the far side of the vessel. In our experience this has not resulted in peritoneal tumors and it does not cause adverse outcomes.

**CRITICAL:** Retract the needle slightly with the needle driver knob to ensure the full bevel is visualized within the vessel lumen.

**CRITICAL:** Adjust the transducer position using the transducer micro-manipulator as needed to visualize the needle tip and bevel.

***Note:*** We have found that the portal vein does not dilate or constrict in response to whole body heating or cooling.

***Note:*** To verify the needle is within the portal vein, the needle can be “bounced” by moving it a couple millimeters up and down (ventral/dorsal with the Z-axis fine adjustment knob on the injection system) and observe that the vessel and surrounding tissue moves with the needle.

***Optional:*** Additionally, the transducer position can be swept several millimeters back and forth (cranio/caudal) with the transducer micro-manipulator to visualize a length of portal vein before and after the needle insertion location.

***Optional:*** An alternative methodology to verify the needle placement within the portal vein is to aspirate a small of volume of blood into the syringe before proceeding with injection.

22. Injection.
  a. Inject 100 μL of saline slowly over approximately 10–15 s.
  b. Leave the needle in place for approximately 30 s after injection.
  c. Retract the needle driver with counterclockwise turns of the knob, then slide the injection system away.

***Note:*** Occasionally hyperechoic dots may be observed in adjacent vessels of the liver during injection as the injectate is perfusing the organ.

23. Monitor for adverse outcomes to determine if prompt humane euthanasia should be considered.
  a. Observe if the portal vein and surrounding liver appears as it did prior to injection.
  b. Pay attention to portal vein structure size and shape.
  c. Assess if any free fluid is around the portal vein or between the liver lobes which would be indicative of a portal vein or bowel rupture, or intraperitoneal injection.

***Note:*** Occasionally a drop of blood is observed externally when the needle is retracted. This is normal and not considered adverse and the mouse can be recovered normally.

24. Retract the transducer, un-tape and recover the mouse in its home cage.
25. Monitor the mouse with a cage-side observation within 2–4 h for signs of distress.

Methods Video S2. Vessels in Liver Identified, related to step #14

Methods Video S3. Dual Synchronized Video Procedure, related to step #12–22

## Expected outcomes

To investigate potential tissue damage in the liver caused by this method, two B6 female mice were injected with saline following the above steps and promptly euthanized. Livers were harvested, fixed in formalin, processed, paraffin-embedded and serially sectioned through the whole block at 20 micron intervals. All 5 micron H&E-stained liver sections were evaluated by a board-certified veterinary pathologist. The needle tract and associated tissue disruption was visualized and consisted mainly of minimal hepatocellular degeneration/necrosis directly adjacent to the needle tract and some minimal hemorrhage. The histopathologic evaluation confirmed that at the level of the portal vein, *there was no evidence of substantial traumatic hemorrhage that would be of concern for the welfare of the mouse or the experimental endpoints*. In conclusion, *a needle tract lesion is anticipated, and the hepatocellular trauma was minimal and not considered limiting for this model*. See Figure 4 “Acute Histology Results” for representative histology images.

**Figure 4:**
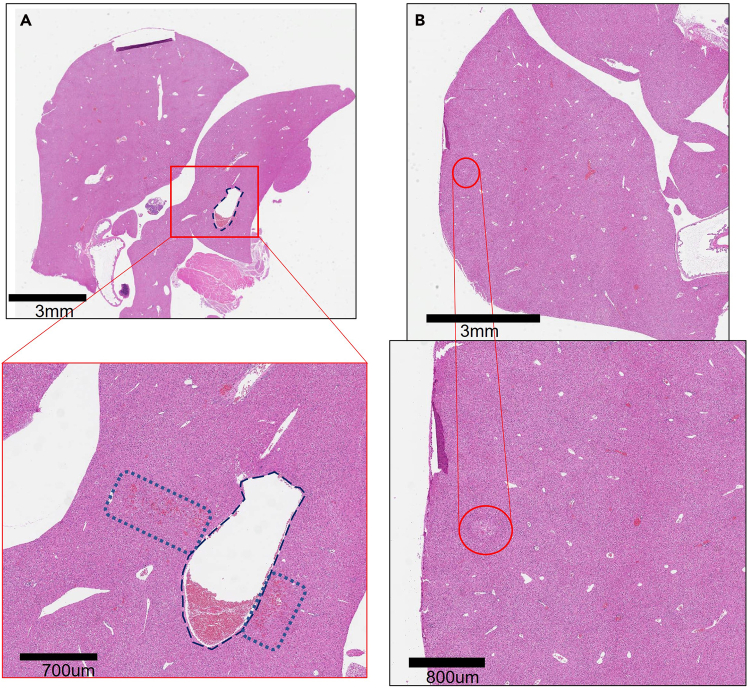
Acute Histology Results Histology results from one mouse liver collected shortly after saline injection. (A) At the level of the portal vein (dashed black lines) minimal increase in red blood cells is seen within the parenchyma at the entry of the needle into the vein and the opposite side (blue dotted boxes). (B) A focal area (red circle) suspected to be a cross-section of a needle tract composed of a center area devoid of hepatocytes, surrounded by at the most 6 hypereosinophilic necrotic hepatocytes. The rim outside is composed of a low number (maximum of 4 layers) of hepatocytes with flocculent cytoplasm (early degeneration) and red blood cells.

To assess the efficacy of the method, cells with concentration ranges of approximately 25 k–100 k in 100 μL of PBS or organoids (dissociated to single cells) at a concentration of 150 K in 100 μL of PBS were used. We have performed this procedure on 71 mice total with four investigators over 5 trials with no adverse outcomes and no reported peritoneal tumors aside from tumors and metastasis in the liver Table1).

**Table 1:** Summary of expected outcomes

Trial #	Sample size	Strain	Sex	Cell line	Injected concentration	Percent rate of success (see expected outcomes section for more info)
1	5	Balb/c	F	MC-26	25 K	80
	10				50 K	
	5				100 K	
2	10	Balb/C	F	MC-26	50 K	80
3	10	B6	F	AKP-tdT CRCOs	150 K	100
4	11	B6	M	AKP-tdT CRCOs	150 K	18
5	5	B6	F	AKP	100 K	100
	5			APC		14
	5			PB2		80
	5			4TO7		40

Our first trial was an exploratory experiment in which 20 Balb/C female mice were injected with mouse colon adenocarcinoma cells (MC-26). 5 mice received 25 K cells, 10 mice received 50 K cells, and 5 mice received 100 K cells. Across all groups 80% of the mice developed liver metastasis while 20% did not develop tumors, and none of the mice developed tumors elsewhere in the peritoneum. Compared to a previously reported intrasplenic surgical injection,^3^ our intrahepatic ultrasound guided injection procedure *led to liver metastasis nodules with more irregular tumor shape* (see Figure 5A)*, and such morphological heterogeneity is histologically similarly to what is observed in the clinic*.^4^ Figure 5 “Expected Outcomes Example” shows in-vivo (Bioluminescence (BLI) and Ultrasound) and ex-vivo (histology and necropsy) results from an injection of mouse colon adenocarcinoma cells (MC-26) at a concentration of 50 k in 100 μL PBS. The diffuse tumor BLI signal across the left side and abdominal area is confirmed to be numerous large tumors in the liver as visualized by ultrasound and corroborated at necropsy. The data in Figure 5 is courtesy of Dr. Liang Hao and Nour Saida Harzallah. A follow up confirmatory study (trial 2) of just the 50 K cell concentration in an additional 10 Balb/C female mice yielded 80% engraftment as well, with the other 20% developing no tumors, and none of the mice developed tumors elsewhere in the peritoneum.

**Figure 5:**
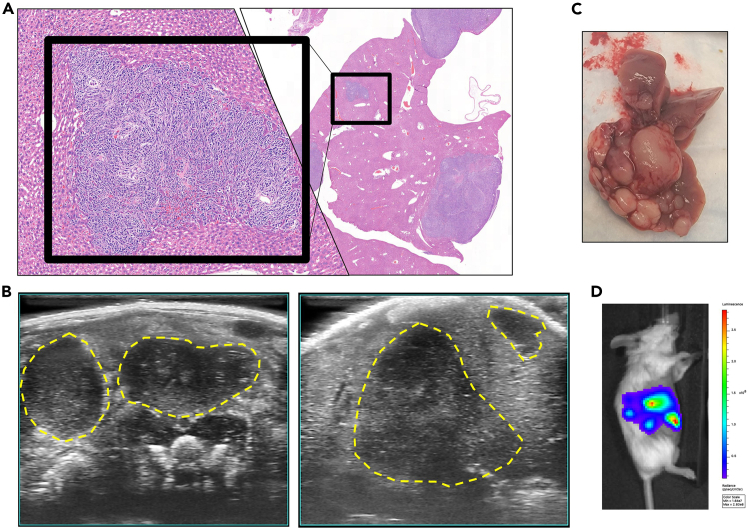
Expected Outcomes Example Representative results from one mouse 6 weeks post injection with 100 μl 50 K MC-26 cells. (A) H&E stained histology image (right side) with magnified portion (left side). (B) Axial 2D ultrasound image of liver area with multiple tumors visualized (dashed yellow lines). (C) Gross necropsy of liver. (D) Bioluminescence image of mouse.

A third trial (trial 3) study with a separate investigator reported 100% engraftment in 10 B6 female mice injected with 150 K colorectal tumor organoids from C57BL/6 mice with mutations in APC, KRAS, and p53 and with fluorescent reporter tdTomato (AKP-tdT CRCOs, dissociated to single cells).

A different trial (trial 4) with the same investigator was conducted on 11 B6 male mice with GFP background. The mice were injected with 150 K AKP-tdT CRCOs (with Diphtheria toxin receptor and expression of GFP) that were dissociated to single cells. Only 2 of the 11 mice were reported to have a successful engraftment, potentially due to the Diphtheria toxin receptor causing an immunogenic rejection in the liver. However, previous orthotopic transplantation in the colon by the investigator showed good engraftment.

A fifth trial (trial 5) with a third investigator sought to probe the metastatic potential of various mouse tumor cells. B6 female mice (n=5 per group) were injected with 100 K cells of the following cell lines:

AKP (colorectal tumor organoids dissociated to single cells).

APC (colorectal adenoma organoids dissociated to single cells).

PB2 (breast tumor cells).

4TO7 (breast tumor cells).

Livers from animals injected with the AKP and APC cell lines were evaluated by fluorescence microscopy ex-vivo at 28- and 35-days post injection. All AKP injected animals developed several metastatic colonies and all animals injected with APC cells were found to only have disseminated cells, but did not show metastatic colonization. Livers from animals injected with the PB2 and 4TO7 cell lines were evaluated by fluorescence microscopy ex-vivo at 28-days post injection For PB2 injected mice, the majority (80%) were found to have an abundant metastatic colony burden. Only 40% of the 4TO7 injected mice had disseminated cells detected in the liver and none of the mice developed metastatic colonization, possibly due to cell growth suppression caused by immunogenicity in the non-syngeneic host.

Future trials will include injections of several liver tumor cell lines, and further evaluations for cell concentration injections up to 400 k for AKP and APC cell lines in healthy young mice as well as in a mouse model of fatty liver disease.

All mice involved in the above trials were housed in static micro-isolator caging with eco-bedding or nestlet enrichment on standard lighting with ad libitum access to food and water. No analgesics have been given for this procedure. No mortality, no distress or poor body condition directly related to the procedure have been observed. Given the aforementioned outcomes, we believe that this ultrasound guided intrahepatic approach offers a refined injection alternative into the portal vein of mice that is in a less complicated location compared to what is reported elsewhere.^2^ The needle insertion through the liver assures no risk of perforating or causing tumor cell engraftment in the pancreas, stomach, kidney, spleen and bowel. Additionally, it is less complicated since it requires very basic organ and blood vessel identification. This approach is appropriate for cells when using the suggested gauge needle, leading to tumors that are distributed amongst the entirety of the liver instead of being isolated to the injection location.^5^ Cells and compounds of interest should be screened for shearing and damage when passed through the 33-gauge needle for this method.

## Limitations

This technique has not been attempted yet in species other than mice, it has not yet been attempted in mice in a diseased condition, it has not been attempted in mice outside of the age and weight range specified, it has only been attempted with cancer cells and organoids as specified in expected outcomes section, and has not been attempted free-hand. This protocol has been implemented using components of the Vevo3100 Imaging System. Specifically, the MX550S transducer was mounted on the transducer micro-manipulator and the image guided injection system for syringe manipulations and has not been replicated in a different experimental setup. In our experience larger diameter needles (smaller numerical gauge size) have been less successful as their bevel is more difficult to fit fully within the lumen of the portal vein by visual verification (step 21), as was also concluded in Walker et al.^5^ During ultrasound a measurement of the portal vein diameter can be made to assist in selecting the appropriate needle bevel length and gauge in cases that may require a different size. We were not able to dilate the portal vein by increasing or decreasing body temperature to allow for easier access for injection. Further limitations of this technique can be determined once the method is applied on a sufficiently large number of mice as well as being attempted by other trained injectionists.

## Troubleshooting

### Problem 1

The transducer micro-manipulator is not available.

### Potential solution

The Vevo Imaging Station 3D Motor can be used in place of the transducer micro-manipulator to adjust the transducer in the Y-axis. Adjustments to the transducer position would be made in the 3D imaging mode motor position panel of the Vevo3100 Imaging System. The 3D motor will need to be initialized prior to the transducer being lowered for imaging.

### Problem 2

Difficulty finding a needle path to the portal vein that does not contain bowel in step 14.

### Potential solution

The imaging platform can be rotated slightly clockwise or counter-clockwise to visualize a different path for the needle that does not contain bowel.

### Problem 3

The length of the needle is only partially visualized in step 17.

### Potential solution

At step 3 when placing the transducer and adjusting the angle of the transducer, take care to ensure these remain in parallel with the injection system as much as possible by eye. Also important for full needle visualization is the injection system holding the needle being in parallel with the transducer. Any slight rotation of the injection system will cause the needle to not appear in plane on the ultrasound image. The injection system rotation lock (Figure 3 label #5) can be released and re-tightened to allow the injection system to reset to the correct positioning. When the lock is loose and the injection system is free to rotate, a groove can be felt at the correct position.

### Problem 4

In step 20 when advancing the needle, the tip of the needle is no longer visible.

### Potential solution

This can occur due to the fine gauge of the needle easily deflecting in the tissue. Ensure you are using the shortest needle length at this gauge as longer needles are more easily deflected. Adjustment of transducer position with the knob on the transducer micro-manipulator should bring the needle bevel back in plane. The mouse position and the needle position should not be adjusted at this time as it could cause damage to abdominal organs.

### Problem 5

In steps 20 and 21 the mouse position shifts slightly laterally when adjusting the transducer position using the transducer micro-manipulator.

### Potential solution

This can occur if the supportive fingers of the right hand (placed in step 19) are removed and used to make the adjustments to the transducer micro-manipulator. Adjustments to the transducer micro-manipulator may need to be made with the left hand to allow for the right hand to remain in the supportive position.

### Problem 6

In step 21, the needle does not puncture the portal vein.

### Potential solution

It is possible the needle has dulled and should be replaced. The vessel walls can be quite elastic and may require quite a bit of force to puncture. Use care to slowly continue to advance the needle to create enough tension on the vessel wall to puncture. We recommend changing the needle any time it appears to have dulled and in compliance with local and institutional regulations.

## Resource availability

### Lead contact

Further information and requests for resources and reagents should be directed to and will be fulfilled by the lead contact, Virginia Spanoudaki (vspan@mit.edu).

### Technical contact

Technical questions on executing this protocol should be directed to and will be answered by the technical contact, Nicole Henning (nhenning@mit.edu).

### Materials availability

This protocol did not generate new unique reagents.

### Data and code availability

This protocol did not generate or analyze datasets or codes.

## Acknowledgments

We would like to thank Kathleen Cormier of the Hope Babette Tang Histology Facility at the Koch Institute for her assistance with the histology sample processing; Dr. Magalie Boucher for her veterinary pathology expertise and Dr. Kelly A. Metcalf Pate for her comments, suggestions, and support, both from the Division of Comparative Medicine at MIT; Dr. Liang Hao of the Department of Biomedical Engineering at Boston University and Nour Saida Harzallah at Harvard Medical School-MIT Health Science and Technology, both formerly of the Bhatia Lab at the Koch Institute for providing expected outcome data and images; and Sarah Elmiligy for the interpretation of the representative bioluminescence results. We also thank Dr. Swagata Goswami and Dr. Jingwei Zhang, postdoctoral associates and Dr. Joseph Charles Sedlak, Harvard/MIT MD-PhD candidate, from the Yilmaz lab at the Koch Institute for providing outcome data. We are grateful to Dr. Aurora Burds for her invaluable technical and collegial support. N.H. would like to acknowledge Patrick Henning for his edits of this protocol.

This work was supported in part by the 10.13039/100016872Koch Institute Support (core) Grant P30-CA014051 from the 10.13039/100000054National Cancer Institute.

## Author contributions

Conceptualization, N.H. and F.R.; investigation, N.H.; methodology, N.H. and F.R.; writing – original draft, N.H.; writing – editing and review, N.H., F.R., and V.S.; Supervision, V.S.

## Declaration of interests

The authors declare no competing interests.
